# Histological Chorioamnionitis and Funisitis as New Risk Factors for Retinopathy of Prematurity: A Meta-analysis

**DOI:** 10.1055/a-2215-0662

**Published:** 2023-12-28

**Authors:** Salma El Emrani, Esther J.S. Jansen, Jelle J. Goeman, Enrico Lopriore, Jacqueline U.M. Termote, Nicoline E. Schalij-Delfos, Lotte E. van der Meeren

**Affiliations:** 1 Department of Ophthalmology, Leiden University Medical Center, Leiden, The Netherlands; 2 Division of Neonatology, Department of Pediatrics, Willem-Alexander Children's Hospital, Leiden University Medical Center, Leiden, The Netherlands; 3 Division of Neonatology, Department of Women and Neonate, Wilhelmina Children's Hospital, University Medical Center Utrecht, Utrecht, The Netherlands; 4 Division of Medical Statistics, Department of Biomedical Data Science, Leiden University Medical Center, Leiden, The Netherlands; 5 Department of Pathology, Leiden University Medical Center, Leiden, The Netherlands; 6 Department of Pathology, Erasmus Medical Center, Rotterdam, The Netherlands

**Keywords:** retinopathy of prematurity, histological chorioamnionitis, funisitis, prematurity, meta-analysis

## Abstract

**Objective** The role of placental inflammation in neonatal morbidities is underestimated due to lack of placental examination. This meta-analysis aims to assess the association between histological chorioamnionitis (HCA) with and without funisitis (FUN) and risk of retinopathy of prematurity (ROP).

**Study Design** Forty-five studies reporting (unadjusted) data on HCA without FUN and HCA with FUN in neonates with ROP were included. Primary outcomes were any stage ROP and severe ROP. Potential confounders explored were gestational age (GA) at birth, birthweight, maternal steroid use, necrotizing enterocolitis, sepsis (suspected/proven) and mechanical ventilation duration.

**Results** Neonates with HCA had increased risk for any stage ROP (odds ratio [OR] 1.8; 95% confidence interval [CI] 1.3–2.4) and severe ROP (OR 1.5; 95% CI 1.2–1.8) compared with neonates without HCA. The rates of any stage ROP (OR 1.8; 95% CI 1.4–2.2) and severe ROP (OR 1.4; 95% CI 1.1–1.6) were higher in neonates with FUN compared with neonates without FUN. Multivariate meta-regression analysis suggests that lower GA increases the effect size between FUN and severe ROP.

**Conclusion** This meta-analysis confirms that presence of HCA and FUN are risk factors for any stage ROP and severe ROP. Structured histological placental examination of HCA and FUN may be a tool to further refine the ROP risk profile.

**Key Points**

This systematic review confirms that HCA is a risk factor for ROP.

This meta-analysis reveals that FUN results in an even higher risk for developing ROP.

Placental examination of HCA/FUN may be a tool to further refine the ROP risk profile.

Retinopathy of prematurity (ROP) is a sight-threatening retinal disorder in premature neonates, accounting for up to 40% of worldwide childhood blindness. 1 This vasoproliferative disorder affects 10 to 25% of neonates born under 32 weeks of gestation, with an even higher incidence of 30 to 70% in neonates with a birthweight (BW) below 1,500 g. 2 The pathophysiology is primarily due to abnormal retinal vessel development and occurs in two phases. The initial phase is induced by hyperoxia exposure and decreased insulin-like growth factor-1 (IGF-1) levels due to placental disruption, followed by decreased vascular endothelial growth factor (VEGF) levels, and results in attenuation and cessation of retinal vessel growth. 1 3 The second phase is characterized by abnormal retinal vessel growth due to increased VEGF concentrations following retinal hypoxia and increasing IGF-1 levels. 1 3

Preterm birth, one of the leading causes of ROP, is associated with chorioamnionitis (CA). 4 Histological chorioamnionitis (HCA) is an acute maternal inflammatory response in the placental membranes, mainly due to ascending microorganisms, while funisitis (FUN) is the fetal inflammatory response of the vessels in the umbilical cord. 5 6 The presence of HCA will not always lead to FUN, whereas the presence of FUN is always equivalent to the presence of HCA. Clinical chorioamnionitis (CCA) has been proven to be unreliable in diagnosing inflammation in the placenta due to high false-positive rates. 7 In most cases, the role of HCA in neonatal morbidities is underestimated due to lack of postpartum placental histological examination. 6

The potential association between HCA and the development of ROP is believed to be a result of proinflammatory cytokines production. 4 8 However, establishing its specific pathophysiology is found to be challenging due to the simultaneous interplay of substantial confounders such as low gestational age (GA) at birth and BW. 4 Additionally, most studies do not differentiate between HCA and FUN, even though the presence of FUN is believed to have more impact on the risk of ROP development. 9

Robust evidence regarding the effect of HCA and especially FUN on the development of ROP is still lacking. Hence, we performed a systematic review and meta-analysis to investigate the association between HCA with and without FUN and the risk of ROP development in premature born neonates to further adjust the risk profile for ROP screening.

## Methods

### Sources

This systematic review was conducted according to Preferred Reporting Items for Systematic Reviews and Meta-Analyses (PRISMA) guidelines and is registered in the International Prospective Register of Systematic Reviews (PROSPERO) (CRD42022346582). 10 The online electronic databases PubMed, Embase, and Cochrane Library were searched up until October 2023 by using combinations of the following keywords: “Placenta,” “Chorioamnionitis,” “Risk Factors,” AND “Retinopathy of Prematurity.” Additionally, various synonyms were added as Medical Subject Headings (MESH) terms and free text words. Lastly, reference lists of key articles were manually reviewed to identify relevant articles missed by PubMed.

### Study Selection

All studies were assessed for eligibility by primarily screening the title and abstract and subsequently evaluating the full text. Studies were eligible for inclusion when (unadjusted) data were reported on HCA with or without FUN in preterm neonates (<37 weeks' GA and/or BW < 2,000 g) with ROP. 11 12 Articles were excluded when the study population consisted entirely of animals or multiple pregnancies due to its confounding effect. 13 Additionally, articles were excluded when placenta reports were unavailable to histologically confirm CA in cases with CCA, which is diagnosed by nonspecific clinical symptoms. 7 Further exclusion criteria were case reports, case series, reviews, editorials, and unavailable full text. To identify eligibility of inclusion, three reviewers (S.E., E.J.S.J., and J.U.M.T.) independently assessed the search results and discrepancies were resolved through discussion.

The primary outcome was ROP. Due to their known potential confounding effect on ROP, the following clinical data were included: GA at birth (weeks), BW (grams) and maternal steroid use, necrotizing enterocolitis (any stage), sepsis (suspected/proven), and mechanical ventilation duration (days). 14 Studies were classified as any stage ROP or severe ROP (defined as ROP ≥ stage 3, Type I ROP [treatment criteria], aggressive [posterior] ROP], plus disease or ROP requiring treatment). 15 HCA was defined as an acute inflammation with presence of neutrophils in the placental membranes (chorion and/or amnion) and chorionic plate. 5 FUN, also known in literature as fetal inflammatory response or acute fetal inflammation, was defined as the presence of neutrophils in the umbilical vessel walls. 5 16

The included neonates were categorized into four groups: HCA and any stage ROP, HCA and severe ROP, FUN and any stage ROP, and FUN and severe ROP. Neonates with HCA were compared with neonates without HCA, while neonates with FUN were compared with neonates without FUN (HCA without FUN or no HCA).

### Quality Assessment

The Newcastle–Ottawa Scale for case–control and cohort studies was used to assess the validity and quality of the included studies. 17 This assessment includes three study aspects: selection (0–4 points), comparability (0–2 points), and exposure/outcome (0–3 points). Scoring was based on the association between HCA/FUN and ROP as the primary outcome. The comparability section relied on GA and one additional potential confounder reported in our study.

### Statistical Analysis

Statistical analyses were performed using R studio for Windows, version 4.2.1 (RStudio, PBC, Boston, MA) with the metafor package, assisted by a statistician (J.J.G.). 18 Data are presented using *n* / *N* (%) and odds ratio (OR) with 95% confidence interval (CI). The OR and 95% CI for the primary outcome were recalculated from unadjusted 2 × 2 table data provided in the studies and combined using a logistic random-effects model due to anticipated heterogeneity. Heterogeneity between studies was evaluated by the *Q* statistics and *I* ^2^ statistics, which is the total variation between studies due to heterogeneity beyond chance. *p* -Values <0.05 were considered statistically significant. Publication bias was assessed through funnel plots, failsafe number, and trim-and-fill function in R studio.

To explain observed between-study heterogeneity, the mean/median difference for continuous variables and the incidence (%) difference for dichotomous outcomes were calculated between the HCA/FUN and no HCA/FUN groups. No distinction was made between the median and mean for continuous variables to include more studies. A univariate meta-regression model was performed to identify variables that potentially influence the effect size of HCA/FUN on ROP. The Bonferroni correction for multiple comparisons was used in univariate analysis, in which a *p* -value of 0.05/number of included variables was considered statistically significant.

Subsequently, variables with significant associations found in univariate analysis were included in multivariate meta-regression analysis. GA and BW are highly correlated and adjusting for both variables in multivariate analysis will undermine the statistical significance of each other. When both GA and BW are significant in univariate analysis, only one variable should be included in multivariate analysis, preferably in combination with the variable small for GA. Since data on small for GA were limited, only the highest significant variable was included to prevent multicollinearity. Data are presented using β-coefficient with 95% CI. Positive β-coefficients indicate that studies with large between-group differences in potential confounders had higher associations between HCA/FUN and ROP, while negative β-coefficients indicate the opposite.

## Results

**Fig. 1:**
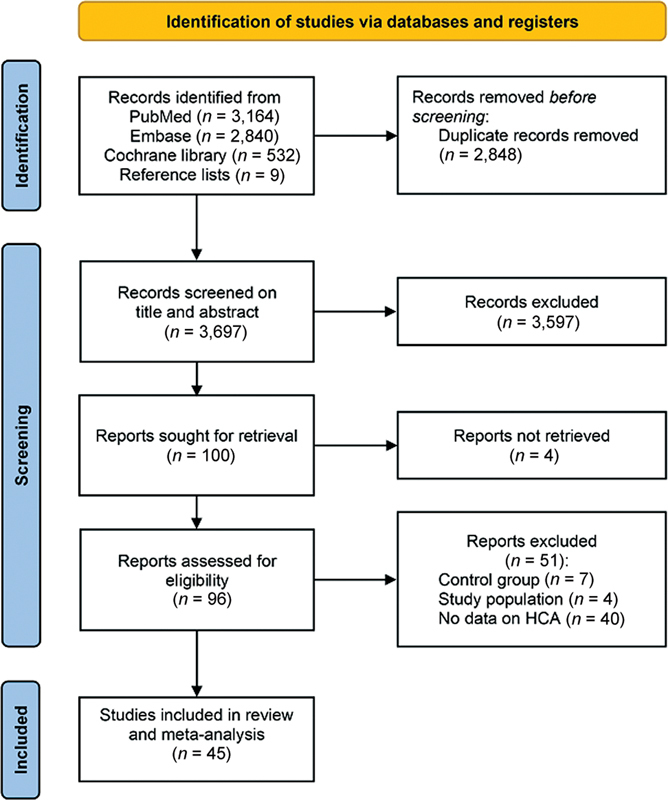
PRISMA flowchart of study inclusion. PRISMA, Preferred Reporting Items for Systematic Reviews and Meta-Analyses.

The search strategy yielded 6,536 articles. Nine additional articles were included from manual search of reference lists for title and abstract screening. After excluding duplicates, 3,697 titles and abstracts were screened. Primary assessment led to exclusion of 3,597 articles based on inclusion and exclusion criteria. After full-text assessment of the remaining 100 articles, 55 articles were excluded, resulting in a total of 45 included articles in this systematic review and meta-analysis ( Fig. 1 ).

### Quality Analysis

Validity and quality assessment of included studies are presented in Supplementary Tables S1–S4 (available in the online version). Most studies ( *n* = 32) had a moderate quality score (6–7 points), while 11 studies received a high quality score (8–9 points), and only 2 studies had a low quality score (0–5 points). Many studies lost points due to absence of confounder adjustment. Some studies that adjusted for both GA and BW in multivariate analysis lost a point since these factors are highly correlated and will undermine the statistical significance of each other. Studies also did not receive points when the definition or reference of ROP and HCA/FUN diagnosis were not reported.

### Histological Chorioamnionitis and Any Stage Retinopathy of Prematurity

**Fig. 2:**
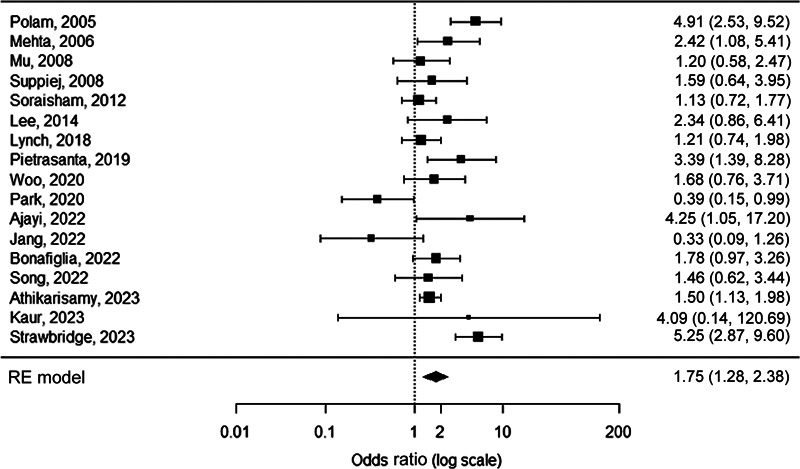
Forest plot of the association between HCA and any stage ROP. Heterogeneity: *Q* = 50.7, *I* ^2^ = 68.5%, *p* < 0.0001. HCA, histological chorioamnionitis; ROP, retinopathy of prematurity; RE, random-effects.

Seventeen studies assessing HCA in neonates with any stage ROP compared with neonates without ROP were included, with the number of sample size ranging from 13 to 912 ( Supplementary Table S1 , available in the online version). 19 20 21 22 23 24 25 26 27 28 29 30 31 32 33 34 35 HCA was detected 1.8 times as frequently in neonates with any stage ROP compared with neonates without ROP (OR 1.8, 95% CI 1.3–2.4, *p* = 0.0004; Fig. 2 ). Neither visual inspection and trim-and-fill number ( *n* = 0) of the funnel plot nor the failsafe number ( *n* = 223, Kendall's tau = 0.07, *p* = 0.7) showed indication of publication bias ( Supplementary Fig. S1 , available in the online version). Significant moderate heterogeneity was observed ( *Q* : 50.7, *I* ^2^ : 68.5%, *p* < 0.0001).

To explore the observed heterogeneity and to analyze the possible influence of potential confounders as moderators on the effect size of HCA on any stage ROP, univariate regression meta-analysis was performed ( Table 1 ). One factor was a significant moderator in univariate meta-regression analysis for the association between HCA and any stage ROP: maternal steroids (β-coefficient 0.05, 95% CI 0.008–0.09, *p* = 0.02).

**Table 1:** Meta-regression regarding the influence of potential confounders on histological chorioamnionitis and any stage retinopathy of prematurity

Univariate	*N*	Estimate	SE	Z-value	*p* -Value	CI lower	CI higher	*R*^2^ (%)	*I*^2^ (%)
Gestational age at birth	8	−0.3920	0.3068	−1.2775	0.2014	−0.9933	0.2094	3.26	74.02
Birthweight	8	−0.0014	0.0012	−1.1361	0.2559	−0.0037	0.0010	1.66	75.11
Maternal steroids	8	0.0482	0.0204	2.3574	**0.0184**	0.0081	0.0882	56.87	53.57
Necrotizing enterocolitis	4	0.0489	0.0728	0.6708	0.5023	−0.0939	0.1916	0.00	43.80
Sepsis	4	0.0381	0.0344	1.1090	0.2674	−0.0293	0.1055	33.56	53.57
Mechanical ventilation	3	−0.0231	0.0735	−0.3144	0.7532	−0.1672	0.1209	0.00	78.90

Abbreviations: SE, standard error; CI, confidence interval.

### Histological Chorioamnionitis and Severe Retinopathy of Prematurity

**Fig. 3:**
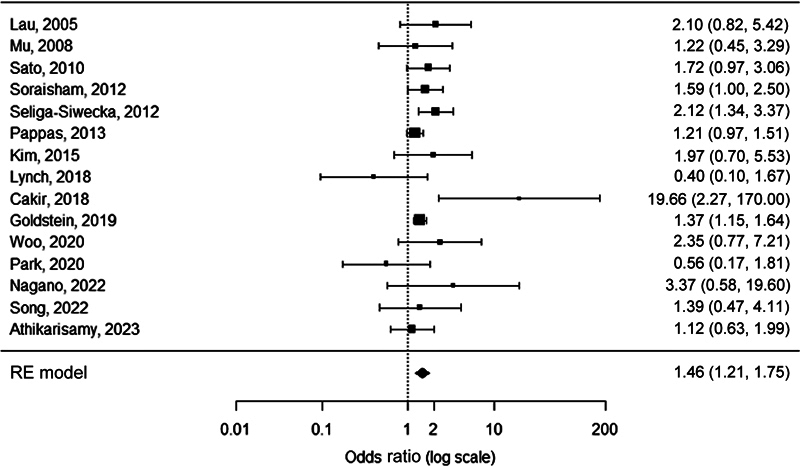
Forest plot of the association between HCA and severe ROP. Heterogeneity: *Q* = 20.2, *I* ^2^ = 30.8%, *p* = 0.1230. HCA, histological chorioamnionitis; ROP, retinopathy of prematurity; RE, random-effects.

Fifteen studies assessing HCA in neonates with severe ROP compared with neonates with no/mild ROP were included, with the number of sample size ranging from 32 to 12,254 ( Supplementary Table S2 , available in the online version). 21 23 25 27 28 32 33 36 37 38 39 40 41 42 43 HCA was detected 1.5 times as frequently in neonates with severe ROP compared with neonates with no/mild ROP (OR 1.5, 95% CI 1.2–1.8, *p* < 0.0001; Fig. 3 ). Publication bias ( Supplementary Fig. S2 , available in the online version) was neither detected in visual inspection and trim-and-fill number ( *n* = 0) of the funnel plot, nor the failsafe number ( *n* = 129, Kendall's tau = 0.1, *p* = 0.5). Low heterogeneity was observed ( *Q* : 20.2, *I* ^2^ : 30.8%, *p* = 0.1230).

While using the Bonferroni correction for multiple comparisons, one factor was a significant moderator in univariate meta-regression analysis for the association between HCA and severe ROP: GA (β-coefficient −0.4, 95% CI −0.7 to −0.04, *p* = 0.03) between the HCA group and non-HCA group ( Table 2 ).

**Table 2:** Meta-regression regarding the influence of potential confounders on histological chorioamnionitis and severe retinopathy of prematurity

Univariate	*N*	Estimate	SE	Z-value	*p* -Value	CI lower	CI higher	*R*^2^ (%)	*I*^2^ (%)
Gestational age at birth	9	−0.3910	0.1777	−2.2006	**0.0278**	−0.7393	−0.0428	74.19	16.81
Birthweight	8	−0.0031	0.0016	−1.9979	0.0457	−0.0062	−0.0001	99.85	0.07
Maternal steroids	9	0.0108	0.0344	0.3132	0.7542	−0.0567	0.0782	0.00	52.15
Necrotizing enterocolitis	8	0.0134	0.0317	0.4238	0.6717	−0.0487	0.0755	0.00	50.26
Sepsis	5	0.0310	0.0153	2.0254	0.0428	0.0010	0.0610	43.36	44.48
Mechanical ventilation	4	−0.0314	0.0563	−0.5588	0.5763	−0.1417	0.0788	0.00	61.30

Abbreviations: SE, standard error; CI, confidence interval.

### Funisitis and Any Stage Retinopathy of Prematurity

**Fig. 4:**
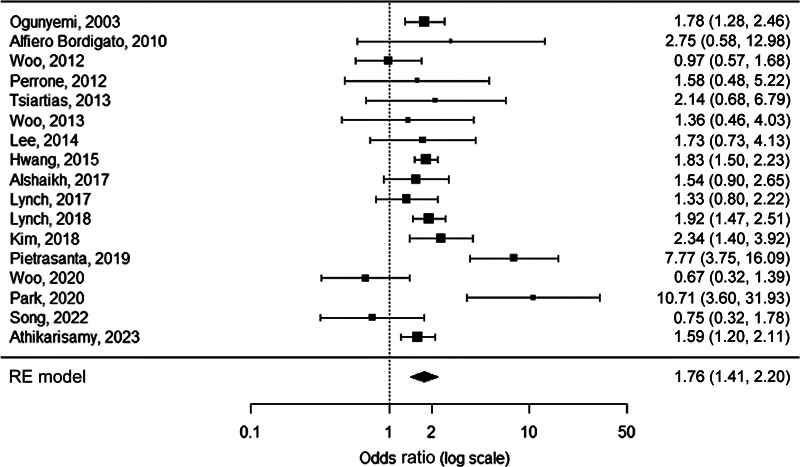
Forest plot of the association between FUN and any stage ROP. Heterogeneity: *Q* = 45.8, *I* ^2^ = 56.1%, *p* < 0.0001. FUN, funisitis; ROP, retinopathy of prematurity; RE, random-effects.

Seventeen studies assessing FUN in neonates with any stage ROP compared with neonates without ROP were included, with the number of sample size ranging from 29 to 2,009 ( Supplementary Table S3 , available in the online version). 24 25 26 27 28 32 33 44 45 46 47 48 49 50 51 52 53 FUN was detected 1.8 times as frequently in neonates with any stage ROP compared with neonates without ROP (OR 1.8, 95% CI 1.4–2.2, *p* < 0.0001; Fig. 4 ). Neither visual inspection and trim-and-fill number ( *n* = 1) of the funnel plot, nor the failsafe number ( *n* = 472, Kendall's tau = 0.04, *p* = 0.8) showed indication of publication bias ( Supplementary Fig. S3 , available in the online version). Significant moderate heterogeneity was observed ( *Q* : 45.8, *I* ^2^ : 65.1%, *p* < 0.0001).

Secondary meta-regression analyses were performed to explore the heterogeneity found in this analysis ( Table 3 ). Two factors were significant moderators in univariate analysis for the association between FUN and any stage ROP: GA at birth (β-coefficient −1.0, 95% CI −1.5 to −0.4, *p* = 0.0004) and sepsis (β-coefficient 0.07, 95% CI 0.02–0.1, *p* = 0.006). In multivariate analysis, no independent factors were found.

**Table 3:** Meta-regression regarding the influence of potential confounders on funisitis and any stage retinopathy of prematurity

	*N*	Estimate	SE	Z-value	*p* -Value	CI lower	CI higher	*R*^2^ (%)	*I*^2^ (%)
**Univariate**									
Gestational age at birth	6	−0.9577	0.2725	−3.5140	**0.0004**	−1.4918	−0.4235	100.00	0.00
Birthweight	6	0.0010	0.0032	0.2986	0.7653	−0.0053	0.0072	0.00	71.13
Maternal steroids	6	−0.0217	0.0273	−0.7953	0.4265	−0.0751	0.0318	16.23	73.71
Necrotizing enterocolitis	4	0.0337	0.1026	0.3289	0.7422	−0.1673	0.2348	0.00	0.00
Sepsis	4	0.0665	0.0241	2.7622	**0.0057**	0.0193	0.1137	100.00	0.00
Mechanical ventilation	2	–	–	–	–	–	–	–	–
**Multivariate**									
Gestational age at birth	4	−0.3234	0.9318	−0.3471	0.7285	−2.1497	1.5029	100.00	0.00
Sepsis		0.0501	0.0530	0.9456	0.3443	−0.0538	0.1540	–	–

Abbreviations: SE, standard error; CI, confidence interval.

### Funisitis and Severe Retinopathy of Prematurity

**Fig. 5:**
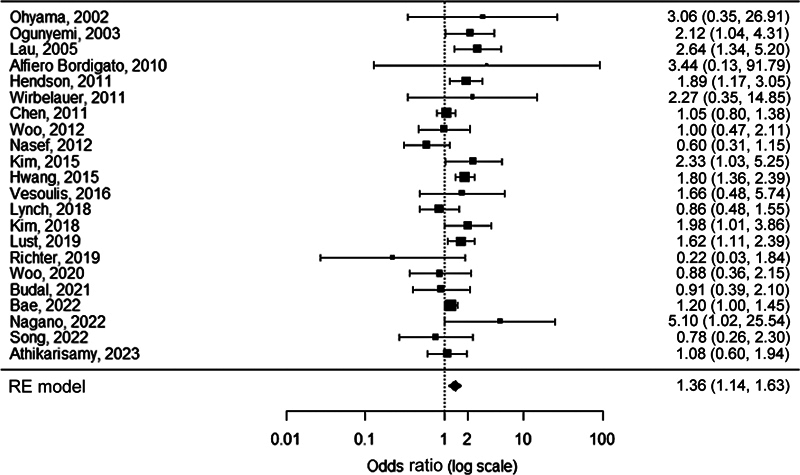
Forest plot of the association between FUN and severe ROP. Heterogeneity: *Q* = 38.4, *I* ^2^ = 45.4%, *p* = 0.0115. FUN, funisitis; ROP, retinopathy of prematurity; RE, random-effects.

Twenty-two studies assessing FUN in neonates with severe ROP compared with neonates with no/mild ROP were included, with the number of sample size ranging from 29 to 2,702 ( Supplementary Table S4 , available in the online version). 25 27 32 33 36 40 43 44 45 46 50 53 54 55 56 57 58 59 60 61 62 63 FUN was detected more frequently in neonates with severe ROP compared with neonates with no/mild ROP (OR 1.4, 95% CI 1.1–1.6, *p* = 0.0008; Fig. 5 ). Publication bias ( Supplementary Fig. S4 , available in the online version) was neither detected in visual inspection and trim-and-fill number ( *n* = 0) of the funnel plot, nor the failsafe number ( *n* = 151, Kendall's tau = 0.004, *p* = 1.0). Significant moderate heterogeneity was observed ( *Q* : 38.4, *I* ^2^ : 45.4%, *p* = 0.0115).

In univariate meta-regression analysis, GA at birth (β-coefficient −0.7, 95% CI −1.2 to −0.3, *p* = 0.001), BW (β-coefficient −0.004, 95% CI −0.007 to −0.001, *p* = 0.007) and maternal steroids (β-coefficient 0.04, 95% CI 0.01–0.07, *p* = 0.009) were found to be significant moderators for the association between FUN and severe ROP ( Table 4 ). GA at birth was more significant compared with BW, which led to BW being omitted from multivariate meta-regression analysis to avoid multicollinearity with GA. In multivariate meta-regression analysis, lower GA at birth (β-coefficient −0.7, 95% CI −1.4 to −0.1, *p* = 0.021) was found to be independently associated with the association between FUN and severe ROP.

**Table 4:** Meta-regression regarding the influence of potential confounders on funisitis and severe retinopathy of prematurity

	*N*	Estimate	SE	Z-value	*p* -Value	CI lower	CI higher	*R*^2^ (%)	*I*^2^ (%)
**Univariate**									
Gestational age at birth	8	−0.7285	0.2213	−3.2923	**0.0010**	−1.1622	−0.2948	100.0	0.00
Birthweight	8	−0.0043	0.0016	−2.7198	**0.0065**	−0.0073	−0.0012	100.0	0.00
Maternal steroids	8	0.0392	0.0149	2.6315	**0.0085**	0.0100	0.0683	87.69	11.42
Necrotizing enterocolitis	5	0.1269	0.0707	1.7952	0.0726	−0.0116	0.2654	100.0	0.00
Sepsis	6	0.0011	0.0491	0.0232	0.9815	−0.0951	0.0973	0.00	64.12
Mechanical ventilation	2	–	–	–	–	–	–	–	–
**Multivariate**									
Gestational age at birth	7	−0.7429	0.3206	−2.3169	**0.0205**	−1.3713	−0.1144	100.00	0.00
Maternal steroids		0.0039	0.0198	0.1962	0.8445	−0.0349	0.0427	–	–

Abbreviations: SE, standard error; CI, confidence interval.

## Summary

Neonates with HCA have a 1.8-fold increased risk for any stage ROP and 1.5-fold increased risk for severe ROP, while neonates with FUN have a 1.8-fold increased risk for any stage ROP and 1.4-fold increased risk for severe ROP. Multivariate regression analysis suggests that lower GA increases the effect size between FUN and severe ROP.

## Discussion

This systematic review and meta-analysis showed that neonates with HCA and/or FUN have an increased risk for any stage ROP and severe ROP. To our knowledge, this is the first meta-analysis with a relatively large number of studies demonstrating that the additional presence of the fetal response results in an even higher risk for developing ROP compared with only the maternal inflammatory response. Meta-regression analysis suggests that lower GA increases the effect size between FUN and severe ROP.

The pathophysiology behind this increased risk of ROP is believed to be due to production of proinflammatory cytokines, which play an important role in retinal angiogenesis, either directly or indirectly by sensitizing the retina to the effects of postnatal oxygen. 64 65 HCA stimulates the production of tumor necrosis factor-α and interleukin (IL)-1, IL-6, and IL-8, which can disrupt the blood–brain barrier and in turn lead to increased plasma protein influx and oligodendroglia damage. 66 67 Increased cytokine levels in the first 72 hours of life have been associated with severe ROP. 68 Additionally, perinatal inflammation induces decreased levels of IGF-1, which inhibit retinal vessel growth and, thus, increase the risk of ROP development. 46 69 70

FUN is the histologic fetal inflammatory response of HCA, and its presence is believed to increase risk of ROP development. 9 However, little is known about FUN since most studies do not differentiate between HCA and FUN. HCA will not always lead to FUN, whereas FUN will always be the result of HCA. Therefore, it is plausible to hypothesize that an additional fetal inflammatory response would increase the risk of ROP compared with only the maternal inflammatory response. Our meta-analyses showed that neonates with FUN indeed had an increased risk for ROP compared with neonates without FUN (HCA without FUN or no HCA), which is based on the most extensive overview of included articles to date.

ROP is a multifactorial disease and multiple hits of antenatal and postnatal inflammation have been found to be associated with ROP. 64 71 72 Studies have shown that sepsis increases the risk of ROP—directly or indirectly—as a consequence of FUN or prematurity. 73 GA < 30 weeks and BW < 1,250 g are the main risk factors in the ROP screening program and could, therefore, partially influence the effect of HCA and FUN. 74 However, GA and BW could also be in the causal pathway, since HCA/FUN can lead to premature birth. The exact pathophysiology remains complex, and several studies have attempted to clarify this mechanism without conclusive evidence. Nevertheless, we have demonstrated that HCA and FUN are associated with ROP and that this association is increased when lower GA is present. Thus, GA is highly suggestive to be an effect modifier.

Three previous meta-analyses have explored the association between CA and ROP and showed opposing results. 4 9 75 Mitra et al reported no association between CA and ROP after adjusting for GA, while Villamor-Martinez et al (OR 1.4, 95% CI 1.1–1.7) and Bahmani et al (OR 1.5, 95% CI 1.0–2.2) showed significant associations. Villamor-Martinez et al also performed a subgroup analysis ( *n* = 2) of FUN and found an increased risk of severe ROP (OR 2.3, 95% CI 1.2–4.3), but not any stage ROP. Notably, our methods of inclusion differed substantially. While the previous meta-analyses investigated the overall effect of CCA and HCA on ROP, we only focused on HCA and the difference between HCA and FUN. Additionally, our meta-analysis included a relatively larger number of studies ( *n* = 17 for any stage ROP, *n* = 22 for severe ROP) with a longer study period (2002–2023) and, therefore, provides stronger evidence.

Remarkably, most studies investigating CA and ROP did not make a distinction between CCA and HCA, even though it has been reported that CCA is unreliable in diagnosing inflammation in the placenta. Studies have shown that intra-amniotic inflammation is only present in around 70% of patients with CCA and should, therefore not be used in scientific publications due to this high false-positive rate. 76 77 Furthermore, recommendations have been proposed to restrict the term CA to pathologic diagnosis, since substantial heterogeneity occurs in the criteria for CCA, and to introduce positive Gram stain, positive amniotic fluid culture and placental histology to the criteria of CA besides isolated maternal fever. 7 Excluding CCA cases provides stronger evidence and may explain the significantly higher odds of ROP in neonates with HCA found in our study.

Several limitations should be considered while interpreting our results. This meta-analysis mainly included retrospective studies, which may have introduced information bias and resulted in limited data on potential confounders. Additionally, severe HCA cases in the HCA group may also have had FUN, since some studies only examined the placenta for HCA. Furthermore, moderate heterogeneity existed between included studies. Nevertheless, our meta-analysis gives the most recent and elaborate overview of the association between HCA and ROP, particularly for the subgroup FUN. Additionally, the differentiation between maternal and fetal inflammatory response demonstrated the importance of recognizing and diagnosing FUN. Lastly, by calculating unadjusted ORs from 2 × 2 tables reported in studies, we have eliminated heterogeneity in confounder adjustment.

In conclusion, this meta-analysis confirms that HCA is a risk factor for any stage ROP and severe ROP. Additionally, it demonstrates the increased odds of any stage and severe ROP in neonates that have also been exposed to FUN. However, ROP is a multifactorial disease and part of the found effect may be influenced by the lower GA found in FUN cases. Future studies should define HCA based on histological placental diagnosis and report additional information on the presence of FUN. Subsequently, meta-regression analysis on potential confounders should be repeated. Nevertheless, our study introduces structured placental examination of HCA and FUN shortly after birth as a potential tool to further refine the ROP risk profile and to possibly tailor treatment interventions in a very early stage to optimize the condition for these vulnerable neonates at risk.
