# Supplementary material for: Histological Chorioamnionitis and Funisitis as New Risk Factors for Retinopathy of Prematurity: A Meta-analysis
Source: Am J Perinatol. 2023 Dec 28;41(Suppl 1):e3264–73. doi: 10.1055/a-2215-0662 (PMC11150066; doi:10.1055/a-2215-0662)
Supplement: Supplementary file 1 — Supplementary Material [file 10-1055-a-2215-0662-s23apr0233.pdf]

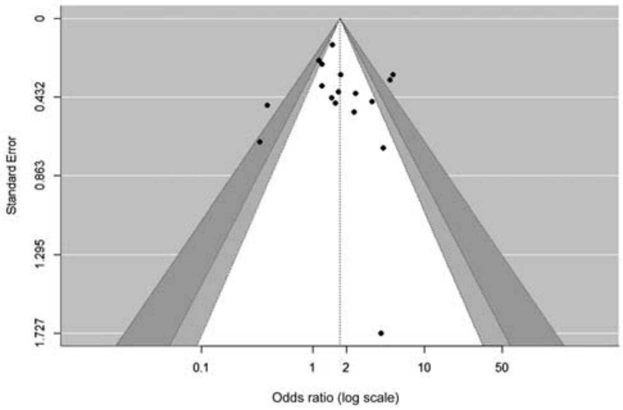

Supplementary Fig. S1 Funnel plot for publication bias assessment of studies reporting on HCA and any stage ROP. HCA, histological chorioamnionitis; ROP, retinopathy of prematurity.

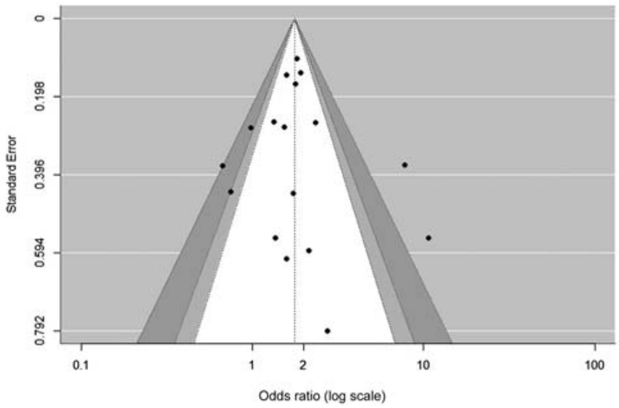

Supplementary Fig. S3 Funnel plot for publication bias assessment of studies reporting on FUN and any stage ROP. FUN, funisitis; ROP, retinopathy of prematurity.

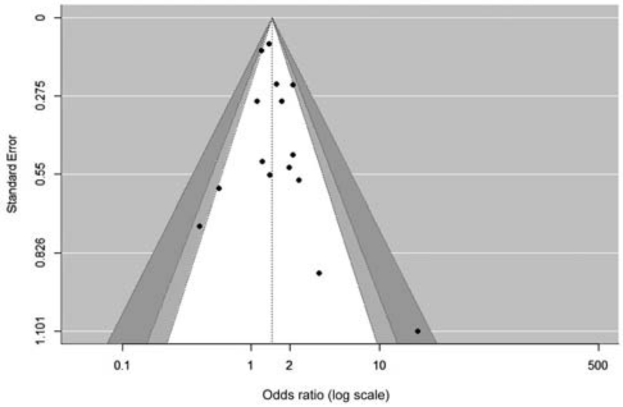

Supplementary Fig. S2 Funnel plot for publication bias assessment of studies reporting on HCA and severe ROP. HCA, histological chorioamnionitis; ROP, retinopathy of prematurity.

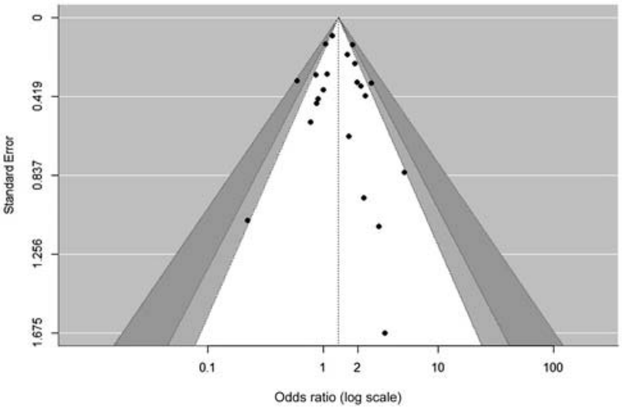

Supplementary Fig. S4 Funnel plot for publication bias assessment of studies reporting on FUN and severe ROP. FUN, funisitis; ROP, retinopathy of prematurity.

 © 2023. The Author(s).

Supplementary Table S1 Study characteristics of any stage retinopathy of prematurity with histological chorioamnionitis

First author, year	Study design	Population	Patients	Definition HCA	Outcome	Validity
Polam, 2005	Retrospective cohort study	United States, 1997 to 2000	HCA: $n=102$<br>No HCA: $n=75$	Five or more neutrophils present per high-power field in the subchorionic space, adjacent chorion, chorionic plate or amnion	HCA<br>❖ Any stage ROP: 81/114 (71.1)<br>❖ No ROP: 21/63 (33.3)	Selection: ★★★★<br>Comparability: –<br>Outcome: ★★
Mehta, 2006	Retrospective cohort study	United States, 1999 to 2001	HCA: $n=64$<br>No HCA: $n=100$	Standard definition for commonly described placental pathological lesions (*wrong reference provided*)	HCA ($p<0.05$)<br>❖ Any stage ROP: 17/30 (56.7)<br>❖ No ROP: 47/134 (35.1)	Selection: ★★★<br>Comparability: –<br>Outcome: ★★
Mu, 2008	Prospective cohort study	Taiwan, 2000 to 2004	HCA: $n=64$<br>No HCA: $n=55$	Presence of inflammatory cell infiltration in the chorion and amnion, and a cloudy/opaque chorionic plate	HCA ($p=1.000$)<br>❖ Any stage ROP: 32/57 (56.1)<br>❖ No ROP: 32/62 (51.6)	Selection: ★★★★<br>Comparability: ★<br>Outcome: ★★
Suppiej, 2008	Prospective cohort study	Italy, 1998 to 2001	HCA: $n=41$<br>No HCA: $n=63$	Presence of acute inflammatory changes on examination of a membrane roll and chorionic plate of the placenta	HCA ($p>0.05$)<br>❖ Any stage ROP: 12/25 (48)<br>❖ No ROP: 29/79 (36.7)	Selection: ★★★★<br>Comparability: –<br>Outcome: ★★★
Soraisham, 2012	Retrospective cohort study with prospective Follow-up	Canada, 2000 to 2006	HCA: $n=197$<br>No HCA: $n=187$	Presence of PMNL infiltration in the placental membranes and chorionic plate	HCA ($p=0.53$)<br>❖ Any stage ROP: 145/278 (52.2)<br>❖ No ROP: 52/106 (49.1)	Selection: ★★★★<br>Comparability: –<br>Outcome: ★★
Lee, 2014	Retrospective cohort study	South Korea, 2005 to 2012	HCA: $n=218$<br>No HCA: $n=121$	Inflammation of the chorioamniotic membrane (acute inflammation in the subchorion/chorion, acute CA, and necrotizing CA)	HCA ($p=0.021$)<br>❖ Any stage ROP: 20/25 (80)<br>❖ No ROP: 198/314 (63.1)	Selection: ★★★★<br>Comparability: ★★<br>Outcome: ★★★
Lynch, 2018	Retrospective cohort study	United States, 2006 to 2016	HCA: $n=82$<br>No HCA: $n=830$	Presence of any stage or grade without involvement of UC or chorionic plate vessels	HCA<br>❖ Any stage ROP: 25/246 (10.2)<br>❖ No ROP: 57/666 (8.6)	Selection: ★★★★<br>Comparability: –<br>Outcome: ★★★
Pietrasanta, 2019	Prospective cohort study	Italy, 2011 to 2014	HCA: $n=73$<br>No HCA: $n=673$	Presence of any stage or grade without involvement of UC or chorionic plate vessels	HCA ($p<0.05$)<br>❖ Any stage ROP: 7/28 (25.0)<br>❖ No ROP: 63/703 (9.0)	Selection: ★★★★<br>Comparability: ★★<br>Outcome: ★★★
Woo, 2020	Retrospective cohort study	South Korea, 2004 to 2018	HCA: $n=128$<br>No HCA: $n=47$	Presence in any five tissue samples (chorionic plate, amnion, UC, or chorion-decidua), of acute inflammatory change	HCA($p=0.196$)<br>❖ Any stage ROP: 40/50 (80.0)<br>❖ No ROP: 88/125 (70.4)	Selection: ★★★★<br>Comparability: –<br>Outcome: ★★★
Park, 2020	Retrospective cohort study with prospective follow-up	South Korea, 2005 to 2014	HCA: $n=58$<br>No HCA: $n=27$	Presence of neutrophils in chorio-decidua, chorionic plate or amnion basement membrane	HCA ($p=0.009$)<br>❖ Any stage ROP: 19/34 (55.9)<br>❖ No ROP: 39/51 (76.5)	Selection: ★★★★<br>Comparability: ★★<br>Outcome: ★★★

 © 2023. The Author(s).

Supplementary Table S1 (*Continued*)

First author, year	Study design	Population	Patients	Definition HCA	Outcome	Validity
Ajayi, 2022	Retrospective cohort study	United States, 2011 to 2016	HCA: $n=35$<br>No HCA: $n=20$	Presence of inflammatory cell infiltration in the chorion or amnion	HCA ($p=0.02$)<br>❖ Any stage ROP: 15/18 (83.3)<br>❖ No ROP: 20/37 (54.1)	Selection: ★★★★<br>Comparability: –<br>Outcome: ★★★
Jang, 2022	Retrospective cohort study	South Korea, 2017 to 2019	HCA: $n=20$<br>No HCA: $n=28$	Presence of inflammation in placental tissue	HCA ($p=0.127$)<br>❖ Any stage ROP: 4/16 (25.0)<br>❖ No ROP: 16/32 (50.0)	Selection: ★★★★<br>Comparability: –<br>Outcome: ★★★
Bonafiglia, 2022	Retrospective cohort study	Italy, 2015 to 2019	HCA: $n=80$<br>No HCA: $n=189$	*No definition or reference given*	HCA ($p=0.06$)<br>❖ Any stage ROP: 23/58 (39.7)<br>❖ No ROP: 57/211 (27.0)	Selection: ★★★<br>Comparability: –<br>Outcome: ★★★
Song, 2022	Retrospective cohort study	South Korea, 2004 to 1019	HCA: $n=97$<br>No HCA: $n=43$	Presence of acute inflammatory change in any tissue sample (UC, chorionic plate, chorio-decidua, or amnion)	HCA ($p=0.389$)<br>❖ Any stage ROP: 27/36 (75.0)<br>❖ No ROP: 70/104 (67.3)	Selection: ★★★★<br>Comparability: –<br>Outcome: ★★★
Athikarisamy, 2023	Retrospective cohort study	Australia, *unreported*	HCA: $n=443$<br>No HCA: $n=435$	Presence of neutrophilic infiltration of the cellular chorion, of the membranes or of the chorionic plate	HCA<br>❖ Any stage ROP: 176/309 (57.0)<br>❖ No ROP: 267/569 (46.9)	Selection: ★★★★<br>Comparability: ★<br>Outcome: ★★★
Kaur, 2023	Prospective case–control study	India, *unreported*	HCA: $n=1$<br>No HCA: $n=0$	Based on histological examination of the placenta (*Amsterdam Placental Workshop Group consensus statement*)	HCA ($p=0.30$)<br>❖ Any stage ROP: 1/6 (16.7)<br>❖ No ROP: 0/7 (0)	Selection: ★★★<br>Comparability: –<br>Outcome: ★★
Strawbridge, 2023	Retrospective cohort study	United States, 2011 to 2021	HCA: $n=71$<br>No HCA: $n=158$	Presence of neutrophilic inflammation of the chorion and amnion	HCA ($p<0.001$)<br>❖ Any stage ROP: 46/87 (52.9)<br>❖ No ROP: 25/142 (17.6)	Selection: ★★★★<br>Comparability: ★<br>Outcome: ★★★

Abbreviations: CA, chorioamnionitis; HCA, histological chorioamnionitis; PMNL, polymorphonuclear leukocyte; ROP, retinopathy of prematurity; UC, umbilical cord.
Data are presented as *n*/*N* (%). Validity scoring was based on the Newcastle-Ottawa Scale and a star represents one point.

 © 2023. The Author(s).

Supplementary Table S2 Study characteristics of severe retinopathy of prematurity with histological chorioamnionitis

First author, year	Study design	Population	Patients	Definition HCA	Outcome	Validity
Lau, 2005	Prospective/ Retrospective cohort study	Canada, 1996 to 1997	HCA: $n=153$<br>No HCA: $n=893$	Presence of PMNLs infiltrating ≥50% of the chorion and/or amnion in submitted section	HCA ($p=0.40$)<br>❖ Severe ROP: 6/23 (26.1)<br>❖ No/mild ROP: 147/1,023 (14.4)	Selection: ★★★★<br>Comparability: –<br>Outcome: ★★★
Mu, 2008	Prospective cohort study	Taiwan, 2000 to 2004	HCA: $n=64$<br>No HCA: $n=55$	Presence of inflammatory cell infiltration in the chorion and amnion, and a cloudy/opaque chorionic plate	HCA ($p=1.000$)<br>❖ Severe ROP: 11/19 (57.9)<br>❖ No/mild ROP: 53/100 (53)	Selection: ★★★★<br>Comparability: ★<br>Outcome: ★★
Sato, 2010	Retrospective cohort study	Japan, 2000 to 2008	HCA: $n=158$<br>No HCA: $n=144$	Presence of neutrophils in chorio-decidua, chorionic plate or amnion basement membrane	HCA ($p<0.01$)<br>❖ Severe ROP: 39/62 (62.9)<br>❖ No/mild ROP: 119/240 (49.6)	Selection: ★★★★<br>Comparability: ★<br>Outcome: ★★
Soraisham, 2012	Retrospective cohort study with prospective follow-up	Canada, 2000 to 2006	HCA: $n=197$<br>No HCA: $n=187$	Presence of PMNL infiltration in the placental membranes and chorionic plate	HCA ($p=0.04$)<br>❖ Severe ROP: 62/104 (59.6)<br>❖ No/mild ROP: 135/280 (48.2)	Selection: ★★★★<br>Comparability: –<br>Outcome: ★★
Seliga-Siwecka, 2012	Prospective cohort study	Poland, 2005 to 2007	HCA: $n=141$<br>No HCA: $n=242$	More than a few scattered neutrophils in the chorionic plate or membranous chorionic connective tissue and/or the amnion	HCA<br>❖ Severe ROP: 51/102 (50.0)<br>❖ No/mild ROP: 90/281 (32.0)	Selection: ★★★★<br>Comparability: ★★<br>Outcome: ★★★
Pappas, 2013	Prospective cohort study	United States, 2006 to 2008	HCA: $n=910$<br>No HCA: $n=1014$	HCA noted on placental pathology report or documented acute/subacute HCA or chronic chorioamnionitis	HCA<br>❖ Severe ROP: 205/416 (49.3)<br>❖ No/mild ROP: 518/1,162 (44.6)	Selection: ★★★★<br>Comparability: ★★<br>Outcome: ★★★
Kim, 2015	Retrospective cohort study	South Korea, 2008 to 2012	HCA: $n=19$<br>No HCA: $n=239$	PMNL infiltration of any grade in the amnion	HCA<br>❖ Severe ROP: 6/50 (12.0)<br>❖ No/mild ROP: 12/185 (6.5)	Selection: ★★★★<br>Comparability: ★<br>Outcome: ★★★
Lynch, 2018	Retrospective cohort study	United States, 2006 to 2016	HCA: $n=82$<br>No HCA: $n=830$	Presence of any stage or grade without involvement of UC or chorionic plate vessels	HCA<br>❖ Severe ROP: 2/51 (3.9)<br>❖ No/mild ROP: 80/861 (9.3)	Selection: ★★★★<br>Comparability: –<br>Outcome: ★★★
Cakir, 2018	Prospective cohort study	Turkey, 2012 to 2015	HCA: $n=35$<br>No HCA: $n=96$	Placental inflammatory immune processes (infectious inflammatory lesions and immune/idiopathic inflammatory lesions)	HCA ($p=0.010$)<br>❖ Severe ROP: 6/7 (85.7)<br>❖ No/mild ROP: 29/124 (23.4)	Selection: ★★★★<br>Comparability: –<br>Outcome: ★★★

 © 2023. The Author(s).

Supplementary Table S2 (*Continued*)

First author, year	Study design	Population	Patients	Definition HCA	Outcome	Validity
Goldstein, 2019	Prospective cohort study	United States, 2007 to 2011	HCA: $n = 971$<br>No HCA: $n = 11283$	Evidence of infection of amniotic sac and fluid or those of the uterine wall	HCA<br>❖ Severe ROP: 162/1597 (10.1)<br>❖ No/mild ROP: 809/10,657 (7.6)	Selection: ★★★<br>Comparability: –<br>Outcome: ★★★
Woo, 2020	Retrospective cohort study	South Korea 2004 to 2018	HCA: $n = 128$<br>No HCA: $n = 47$	Presence in any five tissue samples (chorionic plate, amnion, UC, or chorion-decidua), of acute inflammatory change	HCA($p = 0.125$)<br>❖ Severe ROP: 23/27 (85.2)<br>❖ No/mild ROP: 105/148 (70.9)	Selection: ★★★★<br>Comparability: –<br>Outcome: ★★★
Park, 2020	Retrospective cohort study with prospective follow-up	South Korea, 2005 to 2014	HCA: $n = 58$<br>No HCA: $n = 27$	Presence of neutrophils in chorio-decidua, chorionic plate or amnion basement membrane	HCA ($p = 0.019$)<br>❖ Severe ROP: 8/14 (57.1)<br>❖ No/mild ROP: 50/71 (70.4)	Selection: ★★★★<br>Comparability: –<br>Outcome: ★★★
Nagano, 2022	Retrospective cohort study	Japan, 2019 to 2021	HCA: $n = 21$<br>No HCA: $n = 11$	Histologically confirmed (*Blanc stage* $\geq$ *1*)	HCA ($p = 0.248$)<br>❖ Severe ROP: 9/11 (81.8)<br>❖ No/mild ROP: 12/21 (57.1)	Selection: ★★★★<br>Comparability: –<br>Outcome: ★★★
Song, 2022	Retrospective cohort study	South Korea, 2004 to 2019	HCA: $n = 97$<br>No HCA: $n = 43$	Presence of acute inflammatory change in any tissue sample (UC, chorionic plate, chorio-decidua, or amnion)	HCA ($p = 0.550$)<br>❖ Severe ROP: 15/20 (75.0)<br>❖ No/mild ROP: 82/120 (68.3)	Selection: ★★★★<br>Comparability: –<br>Outcome: ★★★
Athikarisamy, 2023	Retrospective cohort study	Australia, *unreported*	HCA: $n = 443$<br>No HCA: $n = 435$	Presence of neutrophilic infiltration of the cellular chorion, of the membranes or of the chorionic plate	HCA<br>❖ Severe ROP: 26/49 (53.1)<br>❖ No/mild ROP: 417/829 (50.3)	Selection: ★★★★<br>Comparability: ★<br>Outcome: ★★★

Abbreviations: HCA, histological chorioamnionitis; PMNL, polymorphonuclear leukocyte; ROP, retinopathy of prematurity; UC, umbilical cord. Validity scoring was based on the Newcastle-Ottawa Scale and a star represents one point.
Data are presented as *n*/*N* (%).

 © 2023. The Author(s).

Supplementary Table S3 Study characteristics of any stage retinopathy of prematurity with funisitis

First author, year	Study design	Population	Patients	Definition HCA and FUN	Outcome	Validity
Ogunyemi, 2003	Retrospective cohort study	United States, 1992 to 2000	FUN: $n = 254$<br>No FUN: $n = 520$	Included any of the following<br>• Presence of ≥10 PMNLs in the chorion and amnion<br>• FUN (presence of PMNLs in Wharton's jelly or umbilical vessel walls)<br>• Vasculitis (PMNLs in the chorionic or umbilical blood vessel wall)	FUN ($p = 0.001$)<br>❖ Any stage ROP: 92/218 (42.2)<br>❖ No ROP: 162/556 (29.1)	Selection: ★★★★<br>Comparability: –<br>Outcome: ★★
Alfiero Bordigato, 2010	Prospective cohort study	Italy, 2006	FUN: $n = 14$<br>No FUN: $n = 15$	Presence of PMNLs in the amnion, chorio-decidua, UC or chorionic plate	FUN<br>❖ Any stage ROP: 7/11 (63.6)<br>❖ No ROP: 7/18 (38.9)	Selection: ★★★★<br>Comparability: –<br>Outcome: ★★
Woo, 2012	Retrospective cohort study	South Korea, 2004 to 2009	FUN: $n = 97$<br>No FUN: $n = 149$	HCA/FUN: presence of acute inflammatory changes in any of the tissue samples (amnion, chorio-decidua, UC, and chorionic plate)	FUN ($p = 0.926$)<br>❖ Any stage ROP: 32/82 (39.0)<br>❖ No ROP: 65/164 (39.6)	Selection: ★★★★<br>Comparability: –<br>Outcome: ★★★
Perrone, 2012	Prospective cohort study	Italy, 2008 to 2011	FUN: $n = 49$<br>No FUN: $n = 14$	Presence of PMNLs within the UC vessels	FUN ($p > 0.05$)<br>❖ Any stage ROP: 30/37 (81.1)<br>❖ No ROP: 19/26 (73.1)	Selection: ★★★★<br>Comparability: –<br>Outcome: ★★★
Tsiartas, 2013	Cohort study	Czech Republic, 2008 to 2010	FUN: $n = 142$<br>No FUN: $n = 89$	HCA: based on histological grades 3–4 in the chorio-decidua, and/or 3–4 in the chorionic plate, and/or 1–4 in the amnion, and/or 1–4 in the UC	FUN ($p = 0.187$)<br>❖ Any stage ROP: 13/17 (76.5)<br>❖ No ROP: 129/214 (60.3)	Selection: ★★★★<br>Comparability: ★<br>Outcome: ★★
Woo, 2013	Retrospective case–control study	South Korea, 2004 to 2010	FUN: $n = 33$<br>No FUN: $n = 27$	Presence of acute inflammatory changes in tissue samples (amnion, chorio-decidua, UC or chorionic plate)	FUN ($p = 0.542$)<br>❖ Any stage ROP: 12/20 (60.0)<br>❖ No ROP: 21/40 (52.5)	Selection: ★★★★<br>Comparability: ★<br>Outcome: ★★★
Lee, 2014	Retrospective cohort study	South Korea, 2005 to 2012	FUN: $n = 189$<br>No FUN: $n = 148$	Presence of neutrophil infiltration into umbilical vessel walls (umbilical phlebitis/chorionic vasculitis, umbilical arteritis or necrotizing FUN)	FUN ($p = 0.531$)<br>❖ Any stage ROP: 17/25 (68.0)<br>❖ No ROP: 172/312 (55.1)	Selection: ★★★★<br>Comparability: ★★<br>Outcome: ★★★
Hwang, 2015	Prospective cohort study	South Korea, 2013 to 2014	FUN: $n = 586$<br>No FUN: $n = 1423$	Histologically confirmed (*no further definition or reference given*)	FUN ($p < 0.001$)<br>❖ Any stage ROP: 258/686 (37.6)<br>❖ No ROP: 328/1,323 (29.4)	Selection: ★★★<br>Comparability: –<br>Outcome: ★★★
Alshaikh, 2017	Retrospective cohort study	Canada, 2007 to 2010	FUN: $n = 98$<br>No FUN: $n = 184$	Presence of PMNL infiltration in fetal membranes and chorionic plate	FUN ($p = 0.07$)<br>❖ Any stage ROP: 32/76 (42.1)<br>❖ No ROP: 66/206 (32.0)	Selection: ★★★★<br>Comparability: –<br>Outcome: ★★★
Lynch, 2017	Retrospective cohort study	United States, 2008 to 2015	FUN: $n = 194$<br>No FUN: $n = 441$	Presence of PMNL infiltration in placental/fetal membranes	FUN ($p = 0.03$)<br>❖ Any stage ROP: 26/72 (36.1)<br>❖ No ROP: 168/563 (29.8)	Selection: ★★★★<br>Comparability: –<br>Outcome: ★★★

 © 2023. The Author(s).

Supplementary Table S3 (*Continued*)

First author, year	Study design	Population	Patients	Definition HCA and FUN	Outcome	Validity
Lynch, 2018	Retrospective cohort study	United States, 2006 to 2016	FUN: $n = 313$<br>No FUN: $n = 904$	HCA: presence of any stage or grade without involvement of UC or chorionic plate vessels<br>FUN: presence of neutrophils within the muscular walls of veins or arteries in the chorionic plate and/or UC	FUN<br>❖ Any stage ROP: 130/374 (34.8)<br>❖ No ROP: 183/843 (21.7)	Selection: ★★★★<br>Comparability: –<br>Outcome: ★★★
Kim, 2018	Retrospective cohort study	South Korea, 2013 to 2016	FUN: $n = 85$<br>No FUN: $n = 317$	Presence of amniotic fluid infection/ inflammation (neutrophil infiltration into amnion and umbilical vessel walls)	FUN ($p = 0.012$)<br>❖ Any stage ROP: 32/97 (33.0)<br>❖ No ROP: 53/305 (17.4)	Selection: ★★★★<br>Comparability: –<br>Outcome: ★★★
Pietrasanta, 2019	Prospective cohort study	Italy, 2011 to 2014	FUN: $n = 55$<br>No FUN: $n = 731$	HCA: presence of any stage or grade without involvement of UC or chorionic plate vessels<br>FUN: presence of umbilical phlebitis/chorionic vasculitis, umbilical arteritis or necrotizing FUN	FUN<br>❖ Any stage ROP: 13/41 (31.7)<br>No ROP: 42/745 (5.6)	Selection: ★★★★<br>Comparability: ★★<br>Outcome: ★★★
Woo, 2020	Retrospective cohort study	South Korea 2004 to 2018	FUN: $n = 56$<br>No FUN: $n = 119$	Presence of seven neutrophils in the wall of umbilical vessel or Wharton's jelly	FUN ($p = 0.282$)<br>❖ Any stage ROP: 13/50 (26.0)<br>❖ No ROP: 43/125 (34.4)	Selection: ★★★★<br>Comparability: –<br>Outcome: ★★★
Park, 2020	Retrospective cohort study with prospective follow-up	South Korea, 2005 to 2014	FUN: 59<br>No FUN: $n = 26$	Included any of the following<br>• Presence of ≥10 PMNLs in the chorion and amnion<br>• FUN (presence of PMNLs in Wharton's jelly or umbilical vessel walls)<br>• Vasculitis (PMNLs in the chorionic or umbilical blood vessel wall)	FUN ($p = 0.006$)<br>❖ Any stage ROP: 20/34 (58.8)<br>❖ No ROP: 6/51 (11.8)	Selection: ★★★★<br>Comparability: ★★<br>Outcome: ★★★
Song, 2022	Retrospective cohort study	South Korea, 2004 to 2019	FUN: $n = 41$<br>No FUN: $n = 99$	Presence of neutrophils infiltrated the wall of UC vessels and/or Wharton's jelly	FUN ($p = 0.512$)<br>❖ Any stage ROP: 9/36 (25.0)<br>❖ No ROP: 32/104 (30.8)	Selection: ★★★★<br>Comparability: –<br>Outcome: ★★★
Athikarisamy, 2023	Retrospective cohort study	Australia, *unreported*	FUN: $n = 343$<br>No FUN: $n = 535$	Presence of neutrophilic infiltration from the fetal vessels into the umbilical vessels or the chorionic plate vessels	FUN<br>❖ Any stage ROP: 143/309 (46.3)<br>❖ No ROP: 200/569 (35.1)	Selection: ★★★★<br>Comparability: ★<br>Outcome: ★★★

Abbreviations: FUN, funisitis; HCA, histological chorioamnionitis; PMNL, polymorphonuclear leukocyte; ROP, retinopathy of prematurity; UC, umbilical cord. Validity scoring was based on the Newcastle-Ottawa Scale and a star represents one point.
Data are presented as n/N (%).

 © 2023. The Author(s).

Supplementary Table S4 Study characteristics of severe retinopathy of prematurity with funisitis

First author, year	Study design	Population	Patients	Definition HCA and FUN	Outcome	Validity
Ohyama, 2002	Retrospective cohort study	Japan, 1993 to 1996	FUN: $n = 90$<br>No FUN: $n = 53$	Presence of PMNLs in the amnion, chorion, intervillous space, UC, or chorionic plate	FUN ($p > 0.05$)<br>❖ Severe ROP: 5/6 (83.3)<br>❖ No/mild ROP: 85/137 (62.0)	Selection: ★★★★<br>Comparability: –<br>Outcome: ★★
Ogunyemi, 2003	Retrospective cohort study	United States, 1992 to 2000	FUN: $n = 254$<br>No FUN: $n = 520$	Included any of the following<br>• Presence of ≥10 PMNLs in the chorion and amnion<br>• FUN (presence of PMNLs in Wharton's jelly or umbilical vessel walls)<br>• Vasculitis (PMNLs in the chorionic or umbilical blood vessel wall)	FUN ($p = 0.05$)<br>❖ Severe ROP: 16/32 (50.0)<br>No/mild ROP: 238/742 (32.1)	Selection: ★★★★<br>Comparability: –<br>Outcome: ★★
Lau, 2005	Prospective/ Retrospective Cohort study	Canada, 1996 to 1997	FUN: $n = 250$<br>No FUN: $n = 1046$	HCA: Presence of PMNLs infiltrating ≥50% of the chorion and/or amnion in submitted section<br>FUN: Presence of PMNLs inflammation in ≥50% of fetal surface vessels and umbilical vessels in submitted section	FUN ($p = 0.40$)<br>❖ Severe ROP: 14/37 (37.8)<br>No/mild ROP: 236/1,259 (18.7)	Selection: ★★★★<br>Comparability: –<br>Outcome: ★★★
Alfiero Bordigato, 2010	Prospective cohort study	Italy, 2006	FUN: $n = 14$<br>No FUN: $n = 15$	Presence of PMNLs in the amnion, chorio-decidua, UC, or chorionic plate	FUN<br>❖ Severe ROP: 1/1 (100)<br>❖ No/mild ROP: 13/28 (46.4)	Selection: ★★★★<br>Comparability: –<br>Outcome: ★★
Hendson, 2011	Retrospective cohort study with prospective follow-up	Canada, 1997 to 2004	FUN: $n = 225$<br>No FUN: $n = 259$	Acute inflammation with PMNL infiltrating the sub-chorionic fibrin or membrane trophoblast, fibrous chorion, or amnion or/and FIRS (PMNLs invading the UC, chorionic, or umbilical vasculitis)	FUN ($p = 0.011$)<br>❖ Severe ROP: 50/84 (59.5)<br>❖ No/mild ROP: 175/400 (43.8)	Selection: ★★★★<br>Comparability: –<br>Outcome: ★★★
Wirbelauer, 2011	Prospective cohort study	Germany, 2006 to 2007	FUN: $n = 17$<br>No FUN: $n = 54$	Presence of neutrophilic inflammation in the placenta and/or UC	FUN ($p > 0.05$)<br>❖ Severe ROP: 2/5 (40.0)<br>❖ No/mild ROP: 3/54 (22.2)	Selection: ★★★★<br>Comparability: –<br>Outcome: ★★★
Chen, 2011	Prospective cohort study	United States, 2002 to 2004	FUN: $n = 421$<br>No FUN: $n = 641$	The presence of any of the following<br>• Acute inflammation at the chorionic plate of the disc and/or the chorion/decidua<br>• Chorionic plate vasculitis: neutrophilic infiltration of the fetal stem vessels in the chorionic plate	FUN<br>❖ Severe ROP: 122/301 (40.5)<br>❖ No/mild ROP: 299/761 (39.3)	Selection: ★★★★<br>Comparability: ★<br>Outcome: ★★★

 © 2023. The Author(s).

Supplementary Table S4 (*Continued*)

First author, year	Study design	Population	Patients	Definition HCA and FUN	Outcome	Validity
				• Vasculitis: presence of neutrophils in a cord vessel		
Woo, 2012	Retrospective cohort study	South Korea, 2004 to 2009	FUN: $n = 97$<br>No FUN: $n = 149$	HCA/FUN: presence of acute inflammatory changes in any of the tissue samples (amnion, chorio-decidua, UC, and chorionic plate)	FUN ($p = 0.996$)<br>❖ Severe ROP: 13/33 (39.4)<br>❖ No/mild ROP: 84/213 (39.4)	Selection: ★★★★<br>Comparability: –<br>Outcome: ★★★
Nasef, 2012	Retrospective cohort study	Canada, 2007 to 2008	FUN: $n = 95$<br>No FUN: $n = 146$	Presence of inflammation of the chorioamnion or the UC (FUN), chorionic vasculitis, and umbilical phlebitis or vasculitis in the placental pathology	FUN<br>❖ Severe ROP: 16/53 (30.2)<br>❖ No/mild ROP: 79/188 (42.0)	Selection: ★★★★<br>Comparability: –<br>Outcome: ★★★
Kim, 2015	Retrospective cohort study	South Korea, 2008 to 2012	FUN: $n = 31$<br>No FUN: $n = 204$	PMNL infiltration of any grade in the UC	FUN ($p < 0.05$)<br>❖ Severe ROP: 11/50 (22.0)<br>❖ No/mild ROP: 20/185 (10.8)	Selection: ★★★★<br>Comparability: ★<br>Outcome: ★★★
Hwang, 2015	Prospective cohort study	South Korea, 2013 to 2014	FUN: $n = 586$<br>No FUN: $n = 1423$	Histologically confirmed (*no further definition or reference given*)	FUN ($p < 0.05$)<br>❖ Severe ROP: 95/233 (45.5)<br>❖ No/mild ROP: 491/1,776 (27.6)	Selection: ★★★<br>Comparability: –<br>Outcome: ★★★
Vesoulis, 2016	Prospective cohort study	United States, 2012 to 2015	FUN: $n = 23$<br>No FUN: $n = 40$	Based on histological examination of the placenta (*No definition or reference given*)	FUN ($p = 0.53$)<br>❖ Severe ROP: 6/13 (46.2)<br>❖ No/mild ROP: 17/50 (34.0)	Selection: ★★★<br>Comparability: –<br>Outcome: ★★★
Lynch, 2018	Retrospective cohort study	United States, 2006 to 2016	FUN: $n = 313$<br>No FUN: $n = 904$	HCA: presence of any stage or grade without involvement of UC or chorionic plate vessels<br>FUN: presence of neutrophils within muscular walls of veins or arteries in the chorionic plate and/or UC	FUN<br>❖ Severe ROP: 15/65 (23.1)<br>❖ No/mild ROP: 298/1,152 (25.9)	Selection: ★★★★<br>Comparability: –<br>Outcome: ★★★
Kim, 2018	Retrospective cohort study	South Korea, 2013 to 2016	FUN: $n = 85$<br>No FUN: $n = 317$	Presence of amniotic fluid infection/inflammation (neutrophil infiltration into amnion and umbilical vessel walls)	FUN ($p = 0.046$)<br>❖ Severe ROP: 15/46 (32.6)<br>❖ No/mild ROP: 70/356 (19.7)	Selection: ★★★★<br>Comparability: –<br>Outcome: ★★★
Lust, 2019	Retrospective cohort study	United States, 2008 to 2015	FUN: $n = 419$<br>No FUN: $n = 1217$	Based on histological examination of the placenta (*No definition or reference given*)	FUN ($p = 0.013$)<br>❖ Severe ROP: 44/126 (34.9)<br>❖ No/mild ROP: 375/1,510 (24.8)	Selection: ★★★<br>Comparability: ★<br>Outcome: ★★★

(*Continued*)

 © 2023. The Author(s).

Supplementary Table S4 (*Continued*)

First author, year	Study design	Population	Patients	Definition HCA and FUN	Outcome	Validity
Richter, 2019	Retrospective cohort study	The Netherlands, 2012 to 2017	FUN: $n=86$<br>No FUN: $n=139$	*No definition or reference given*	FUN ($p=0.116$)<br>❖ Severe ROP: 1/8 (12.5)<br>❖ No/mild ROP: 85/217 (39.2)	Selection: ★★★<br>Comparability: –<br>Outcome: ★★★
Woo, 2020	Retrospective cohort study	South Korea, 2004 to 2018	FUN: $n=56$<br>No FUN: $n=119$	Presence of seven neutrophils in the wall of umbilical vessel or Wharton's jelly	FUN ($p=0.774$)<br>❖ Any stage ROP: 8/27 (29.6)<br>❖ No ROP: 48/148 (32.4)	Selection: ★★★★<br>Comparability: –<br>Outcome: ★★★
Budal, 2021	Prospective observational study	Norway, 2010 to 2018	FUN: $n=61$<br>No FUN: $n=40$	Presence of PMNL infiltration in the placenta, extraplacental membranes and UC	FUN<br>❖ Severe ROP: 20/34 (58.8)<br>❖ No/mild ROP: 41/67 (61.2)	Selection: ★★★★<br>Comparability: ★★<br>Outcome: ★★★
Bae, 2022	Prospective cohort study	South Korea, 2013 to 2017	FUN: $n=1276$<br>No FUN: $n=1426$	Presence of acute inflammatory changes in the choriodecidua, amnion, UC and chorionic plate	FUN ($p=0.053$)<br>❖ Severe ROP: 277/544 (50.9)<br>❖ No/mild ROP: 999/2,158 (46.3)	Selection: ★★★★<br>Comparability: –<br>Outcome: ★★★
Nagano, 2022	Retrospective cohort study	Japan, 2019 to 2021	FUN: $n=10$<br>No FUN: $n=22$	Histologically confirmed (*Blanc stage* $\geq$ *1*)	HCA ($p=0.056$)<br>❖ Severe ROP: 6/11 (54.5)<br>❖ No/mild ROP: 4/21 (19.0)	Selection: ★★★★<br>Comparability: –<br>Outcome: ★★★
Song, 2022	Retrospective cohort study	South Korea, 2004 to 2019	FUN: $n=41$<br>No FUN: $n=99$	Presence of neutrophils infiltrated the wall of UC vessels and/or Wharton's jelly	FUN ($p=0.649$)<br>❖ Severe ROP: 5/20 (25.0)<br>❖ No/mild ROP: 36/120 (30.0)	Selection: ★★★★<br>Comparability: –<br>Outcome: ★★★
Athikarisamy, 2023	Retrospective cohort study	Australia, *unreported*	FUN: $n=343$<br>No FUN: $n=535$	Presence of neutrophilic infiltration from the fetal vessels into the umbilical vessels or the chorionic plate vessels	FUN<br>❖ Severe ROP: 20/49 (40.8)<br>❖ No/mild ROP: 323/829 (39.0)	Selection: ★★★★<br>Comparability: ★<br>Outcome: ★★★

Abbreviations: FIRS, fetal inflammatory response syndrome; FUN, funisitis; HCA, histological chorioamnionitis; PMNL, polymorphonuclear leukocytes; ROP, retinopathy of prematurity; UC, umbilical cord. Validity scoring was based on the Newcastle-Ottawa Scale and a star represents one point.
Data are presented as *n*/*N* (%).

 © 2023. The Author(s).
